# Antibodies Against the Clock Proteins Period and Cryptochrome Reveal the Neuronal Organization of the Circadian Clock in the Pea Aphid

**DOI:** 10.3389/fphys.2021.705048

**Published:** 2021-07-02

**Authors:** Francesca Sara Colizzi, Katharina Beer, Paolo Cuti, Peter Deppisch, David Martínez Torres, Taishi Yoshii, Charlotte Helfrich-Förster

**Affiliations:** ^1^Neurobiology and Genetics, Theodor-Boveri-Institute, Biocenter, University of Würzburg, Würzburg, Germany; ^2^Institute for Integrative Systems Biology (I2SysBio), University of Valencia and CSIC, Valencia, Spain; ^3^Graduate School of Natural Science and Technology, Okayama University, Okayama, Japan

**Keywords:** aphids, circadian clock, cryptochrome, period, hemiptera, insects, photoperiodism

## Abstract

Circadian clocks prepare the organism to cyclic environmental changes in light, temperature, or food availability. Here, we characterized the master clock in the brain of a strongly photoperiodic insect, the aphid *Acyrthosiphon pisum*, immunohistochemically with antibodies against *A. pisum* Period (PER), *Drosophila melanogaster* Cryptochrome (CRY1), and crab Pigment-Dispersing Hormone (PDH). The latter antibody detects all so far known PDHs and PDFs (Pigment-Dispersing Factors), which play a dominant role in the circadian system of many arthropods. We found that, under long days, PER and CRY are expressed in a rhythmic manner in three regions of the brain: the dorsal and lateral protocerebrum and the lamina. No staining was detected with anti-PDH, suggesting that aphids lack PDF. All the CRY1-positive cells co-expressed PER and showed daily PER/CRY1 oscillations of high amplitude, while the PER oscillations of the CRY1-negative PER neurons were of considerable lower amplitude. The CRY1 oscillations were highly synchronous in all neurons, suggesting that aphid CRY1, similarly to Drosophila CRY1, is light sensitive and its oscillations are synchronized by light-dark cycles. Nevertheless, in contrast to *Drosophila* CRY1, aphid CRY1 was not degraded by light, but steadily increased during the day and decreased during the night. PER was always located in the nuclei of the clock neurons, while CRY was predominantly cytoplasmic and revealed the projections of the PER/CRY1-positive neurons. We traced the PER/CRY1-positive neurons through the aphid protocerebrum discovering striking similarities with the circadian clock of *D. melanogaster*: The CRY1 fibers innervate the dorsal and lateral protocerebrum and putatively connect the different PER-positive neurons with each other. They also run toward the pars intercerebralis, which controls hormone release *via* the neurohemal organ, the corpora cardiaca. In contrast to *Drosophila*, the CRY1-positive fibers additionally travel directly toward the corpora cardiaca and the close-by endocrine gland, corpora allata. This suggests a direct link between the circadian clock and the photoperiodic control of hormone release that can be studied in the future.

## Introduction

Living beings evolved in a cyclic environment in which many factors, such as light, temperature, humidity, and food availability oscillate in a daily 24-h rhythm. Endogenous circadian clocks help organisms to predict and adapt to these changes. In most animals, a master clock in the brain is synchronized *via* retinal and extra-retinal photoreceptors with the 24 h light-dark cycle and controls behavior, physiology, and metabolism in a rhythmic manner *via* different neuronal and hormonal output pathways (Pilorz et al., 2018; King and Sehgal, 2020). In addition to controlling daily rhythms, the master clock is also thought to serve as internal reference for measuring day length (Bünning, 1936; Shim et al., 2017; Wood et al., 2020; Saunders, 2021). The latter capability is important to anticipate and prepare in advance for seasonal changes in the environment, also known as photoperiodic response. The involvement of the circadian clock in photoperiodic responses is established in various organisms from plant to mammals but, in insects, it is still an open question with intense controversy (Bradshaw and Holzapfel, 2017). One reason of this controversy lies in the problem that the circadian clocks of strongly photoperiodic insects and their connections to the photoperiodic control centers have only been elucidated in few insects (Hamanaka et al., 2005; Shiga and Numata, 2009; Ikeno et al., 2014; Yasuyama et al., 2015). The aim of this study was to characterize the neuronal network of the circadian clock in the brain of another strongly photoperiodic insect, the pea aphid *Acyrthosiphon pisum*.

The pea aphid drastically changes its reproductive strategy over the year, and these changes are induced by the photoperiod (Marcovitch, 1924; Lees, 1959; Hardie and Vaz Nunes, 2001). In spring and summer, the population is composed only of females which reproduce in a viviparous-parthenogenetic way. In autumn, when the day length becomes shorter, females produce sexual generations of males and females, which can mate and produce fertilized eggs. The egg is the only aphid morph, which is able to survive harsh winter conditions. In spring, the eggs hatch and new parthenogenetic females are born. Here, we studied the circadian clock of viviparous-parthenogenetic females under long-day conditions.

Circadian clocks are generally based on molecular transcriptisonal and translational feedback loops, which are interconnected and result in a rhythmic expression of genes (reviewed by Pilorz et al., 2018; Beer and Helfrich-Förster, 2020). In *Drosophila melanogaster*, the clock of which is best studied among insects, the fundamental feedback loop includes the genes *period* (*PER*), *timeless* (*tim*), *clock* (*CLK*), *cycle* (*CYC*), and their respective protein products PER, TIM, CLK, and CYC. CLK and CYC form heterodimers that work as positive transcription factors for *per*, *tim*, and other genes. PER and TIM proteins form themselves heterodimers which enter in the nucleus inhibiting their own transcription by binding the CLK–CYC complex. For the synchronization with the external light-dark cycle, the *Drosophila* clock uses photoreceptors in the compound eyes and the Hofbauer-Buchner eyelets as well as the blue-light photoreceptor Cryptochrome-1 (CRY1), which is located in the circadian clock neurons themselves (reviewed by Helfrich-Förster, 2020). When activated by light, CRY1 leads to the degradation of TIM, which resets the clock in a daily manner. Mammals and other insects, including aphids, possess a different form of cryptochrome that appears light insensitive, takes the role of TIM in the core clock and is called Cryptochrome-2 (CRY2 or mammalian-CRY; Zhu et al., 2005; Rubin et al., 2006; Yuan et al., 2007; Cortés et al., 2010). While the honeybee *Apis mellifera* and the beetle *Tribolium castaneum* possess only CRY2 (Zhu et al., 2005; Rubin et al., 2006), mosquitoes, butterflies, and aphids have CRY1 and CRY2 (Yuan et al., 2007; Cortés et al., 2010). Aphids appear even to possess two copies of CRY2 (called *CRY2-*1 and *CRY2-*2) as revealed by bioinformatics analysis (Cortés et al., 2010). Nevertheless, it is unclear whether these have the same role as mammalian-CRY since aphids also possess TIM that is absent in mammals (Barberà et al., 2017).

More recent *in situ* hybridizations and qPCRs of clock transcripts showed that *per* and *tim* are transcribed in the dorsal and lateral protocerebrum of *A. pisum* and oscillate in a daily manner (Barberà et al., 2017). However, nothing is known about the clock proteins and the anatomy of the neuronal clock network of aphids. In this paper, we characterize the spatial and temporal expression of the clock proteins PER and CRY. We found that the aphid clock shares homologies with *Drosophila’s* clock but has also its own peculiarities. As true for *D. melanogaster*, most of the aphid clock neurons are situated in the dorsal and lateral protocerebrum and about half of them express CRY. However, aphids also show a cluster of clock proteins expressing cells in the distal optic lobe (the lamina). Moreover, we show that CRY is present in the entire neurites as true for *D. melanogaster*. Nevertheless, CRY is not degraded by light suggesting clear difference to *Drosophila* CRY. Finally, tracing the CRY-positive neurons in *A. pisum*, we found that some axons project to the pars intercerebralis and others to the corpora cardiaca/allata complex, the neuroendocrine system that secretes insulin-like peptides (ILPs) into the circulation (Nässel and Broeck, 2016) and produces and secretes Juvenile Hormone (Taguchi et al., 2017). Our findings unravel putative anatomical links between the circadian clock and hormonal centers, which may lead to a seasonally dependent release of hormones and can be studied in detail in the future.

## Materials and Methods

### Aphid Strains

In this study, two lines of *A. pisum* were used: the HOR line was collected in Würzburg (Germany) and kindly provided by Jens Joschinski (Ghent University, Belgium) and the GR line was collected in Gallur (Spain) and kindly provided by David Martínez-Torres. Both lines gave similar results, and therefore, we did not distinguish between them further in the manuscript. Aphids were reared as parthenogenetic clones on *Vicia faba* plants in climate chambers (Sanyo/Panasonic MLR-352H series; 18 ± 0.5°C, 80 ± 10% RH) under a light regime LD 16:8.

### Antibodies

#### Anti-PER

Two different newly generated polyclonal antibodies against *A. pisum* PER were used in this study. The first one was generated in rats by the Li International Company (Denver CO, United States. Product ID: AB002865) and directed against residues 969–1018 of *A. pisum* PER that correspond to the peptide sequence DGDECYISIGKNKRRRIDFCRMAMIYEEDALIPPPPTSPRKLSKSDQSTS. The second PER antibody was generated in guinea pigs against recombinant GST-fused *A. pisum* PER (residues 918–1,018) expressed in *Escherichia coli.* The relevant sequence is PDLIYRYQMNYGDVNEVLRKDINTLNTFTQPMLVNEQFKQLCVEIDVNGSSKTSYFEDGTSSSSDGDECYISIGKNKRRRIDFCRMAMIYEEDALIPPPPTSPRKLSKSDQSTS. The corresponding DNA was synthesized after codon optimization for bacterial expression (GenScript, New Jersey, United States). The synthesized DNA was cloned into the pGEX-6p-1 vector (GE Healthcare, Chicago, United States) using the In-Fusion cloning method (Takara, Japan). The GST-*A. pisum* PER was expressed in *E. coli* B21 (BioDynamics Laboratory Inc., Japan), and the purified protein was used for immunization (Scrum Inc., Japan).

#### Anti-CRY

To localize CRY expressing cells, we used a polyclonal antibody against *D. melanogaster* CRY1. The antibody was generated by immunizing rabbits with a full-length *Drosophila* CRY1 protein, fused to a histidine tag and purified from *E. coli* extracts. The antibody was first described by Yoshii et al. (2008), and its specificity was proofed in *cry* null mutant cry^OUT^ flies (Yoshii et al., 2008). This antibody was kindly provided by the Takeshi Todo (Osaka, University, Osaka, Japan). Since the sequences of aphid CRY1 and CRY2s are rather similar we anticipated that this polyclonal *Drosophila* antibody may recognize both aphid CRYs.

#### Anti-PDH

Since the Pigment-Dispersing Factor (PDF) is an important neuropeptide in the circadian system of the majority of insects, we used a polyclonal antibody against the crab Pigment-Dispersing Hormone (PDH) to reveal PDF-positive neurons in the aphid brain (Dircksen et al., 1987). Anti-PDH is superior to the many different anti-PDF antisera that are available because it reliable detects PDFs and PDHs of all arthropods (Mayer et al., 2015). This antibody was kindly provided by the Heinrich Dircksen (Stockholm University, Sweden).

### Western Blots

To verify the specificity of the CRY1 antibody in aphids, we extracted the proteins from aphid heads and checked *via* Western blot analysis whether it binds to a single protein band at 64 kDa (corresponding to the CRY1 mass in *A. pisum*) or also to the protein band at 59 kDa (corresponding to the CRY2 mass). Aphids were entrained at 18°C either in LD 16:8 or in constant light. Twenty-five adults per each condition were collected at ZT21, when a high amount of protein expression was expected according to the studies in *D. melanogaster*. Right after collection, aphids were decapitated and the heads were suspended in the protein extraction buffer (7× “complete, Mini, EDTA-free” protease inhibitor mix from Roche). Heads then were opened in the solution with plastic pestles and the lysate was cleared spinning down for 6 min at 4°C and 14,800 rpm. Proteins then were denaturated by cooking at 95°C for 5 min. The proteins were separated using SDS-PAGE 12% gel and blotted on a Protran^®^ nitrocellulose membrane from *Whatman*^®^. The membrane was blocked for 2 h using Odyssey^®^ Blocking Buffer by LI-COR in a 1:1 ratio with a Tris-buffered saline (TBS). The membrane was incubated with the primary antibody solution [5% Odyssey^®^ Blocking Buffer, anti-CRY1 rabbit 1:8000, NaN_3_ 1:5000, in TBST (TBS containing 0.5% Tween 20^®^)] overnight at 4°C. After washing 4 × 10 min with TBST, membranes were incubated with the secondary antibody solution (5% Odyssey^®^ Blocking Buffer, Alexa Fluor^®^ 800 goat anti-rabbit (Thermo Scientifc), NaN_3_ 1:5000) for 2 h at room temperature. Finally, the membrane was analyzed with an infrared fluorescence laser scanner (Odyssey by LI-COR).

### Dissection and Immunohistochemical Procedure

#### Fluorescent Immunostaining With Anti-PER and Anti-CRY at ZT21

For immunohistochemical staining, adult aphids were collected at ZT21. Aphids were fixed in 4% paraformaldehyde in phosphate buffered saline (PBS) containing 0.5% Triton-X100 (PBST) on a tube rotator for 4 h in darkness at room temperature. After fixation, aphids were washed 3 × 10 min in PBS and the brains were dissected in PBS. Right after preparation, brains were incubated in the blocking solution (5% normal goat serum in PBST 0.5%) for 2 h at room temperature or overnight at 4°C. Brains were subsequently incubated in the primary antibody solution (5% normal goat serum in PBST, anti-PER rat 1,3,000 or anti-PER guinea pig 1:1000, anti-CRY1 1:1000, NaN_3_ 1:5000) for two nights at 4°C. After washing the brains 6 × 10 min in PBST, they were incubated in the secondary antibody solution [5% normal goat serum in PBST, Alexa Fluor 488 goat anti-rat or goat anti-guinea pig 1:200; Alexa Fluor 635 goat anti-rabbit 1:200 (Thermo Scientifc)] for 4 h in darkness at room temperature. Brains were then washed 4 × 10 min at room temperature in PBST and rinsed two more times in PBS. To test the nuclear localization of PER, we performed co-immunostainings with anti-PER and DAPI (4,6-diamidino-2-phenylindol), which intercalates in the double-stranded DNA. We incubated the brains in 2.5 μg/ml DAPI in PBST after washing out the secondary antibody solution. Brains were finally put on a specimen slide and embedded in a drop of Vectashield Antifade mounting medium (Vector Laboratories, Burlingame, CA). The slides were stored at 4°C in darkness until scanning.

#### Time Course Experiment

Adult aphids were first entrained for at least a week in LD (16:8 h) and then collected every 4 h for seven times starting at ZT0. Ten aphids were collected per time point. For each collection, the same procedure of the “Immunostaining at ZT21” (see previous paragraph) was applied. The only difference came with the incubation in the secondary antibody solution, which lasted one night at 4°C plus 4 h at room temperature, always in darkness.

#### Fluorescent Immunostaining With Anti-PDH at ZT21

Ten adult aphids were dissected and immunostained at ZT21 as described above. The anti-PDH antibody was applied at a dilution of 1:3000.

### Evaluation of Immunostainings

#### Immunostainings at ZT21

The antibody stainings were visualized with a Leica TCS SPE confocal microscope (Leica, Wetzlar, Germany). We used a 20-fold glycerol immersion objective (ACS APO Leica Microsystem, Wetzlar, Germany) and obtained stacks of 2 μm and 1,048,576 pixels. We sequentially used two different diode laser lines double (488 and 635 nm) immunolabeling to excite the fluorophores of the secondary antibodies. In the PER-DAPI immunostainings, the excitation wavelength of the lasers was, respectively, 488 and 405 nm. The obtained confocal stacks were maximum projected and analyzed with Fiji ImageJ (Schindelin et al., 2012). Only contrast, brightness, background correction, and color scheme adjustments were applied to the confocal images. The 3D reconstructions of CRY-positive neurons were generated using two plug-ins of Fiji ImageJ (Schindelin et al., 2012): Simple Neurite Tracer and 3D Viewer.

#### Time Course Experiment

The antibody staining was visualized with a Leica TCS SP8 confocal microscope (Leica Microsystems, Wetzlar, Germany). We used a 20-fold glycerol immersion objective (HC PL APO, Leica Microsystem, Wetzlar, Germany) and obtained stacks of 2 μm and 1,048,576 pixels. We sequentially used two different white-light lasers (488 and 635 nm) to excite the fluorophores of the secondary antibodies. The obtained confocal stacks were maximum projected and analyzed with Fiji ImageJ (Schindelin et al., 2012). Only contrast, brightness, background correction, and color scheme adjustments were applied to the confocal images.

### Quantification and Statistical Analysis of the Time Course Experiment

The quantification of PER and CRY levels was performed measuring the staining intensity in the nuclear and cytoplasmic area, respectively, using the software Fiji (Schindelin et al., 2012). The background fluorescence was subtracted from the mean intensity of each cluster. To assess for significant differences between the time points of each cluster we used the Kruskal–Wallis-H Test. To assess for the rhythmicity of the curves, we used the JTK_CYCLE test (Hughes et al., 2010). Both statistical tests were performed in R version 3.6.3.

## Results

### The *Drosophila*-CRY Antibody Most Likely Recognizes Aphid CRY1, but in Contrast to *Drosophila* CRY1, Aphid CRY1 Is Not Degraded by Light

**Figure 1:**
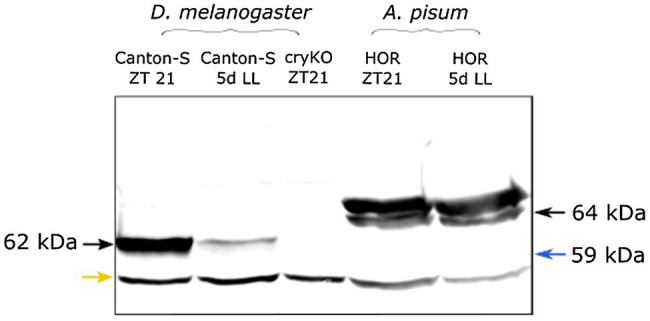
The Drosophila CRY1 antibody appears to detect aphid CRY1. As expected, the Drosophila anti-CRY1 recognizes one single band in the Drosophila head extract. Under constant light (5d LL), the signal decreased because of CRY1 degradation by light. In the cryKO mutant, the signal was absent. The same antibody detected two bands in the aphid head extract [one of them is at the expected height of aphid CRY1 (64 kDa) and the other is at 65 kDa]. We do not see any band at 59 kDa (blue arrow) where we would have expected CRY2. When aphids are reared for 5 days under constant light (5d LL), the signal does not decrease, suggesting that the amount of CRY protein does not change. The lower band in all lanes likely results from unspecific binding (yellow arrow).

Bioinformatic analysis made by Cortés et al. (2010) revealed that the *A. pisum* genome possesses the *Drosophila*-type *cry* (insect-type 1 or *cry*1) and two copies of the mammalian-type *cry* (insect-type 2 or *cry*2) of cryptochrome genes (called *cry*2*-*1 and *cry*2*-*2). The *Drosophila* CRY1 protein has 48% amino acid sequence identity with the aphid CRY1 and 39% of amino acid sequence identity with both aphid CRY2-1 and CRY2-2, making it possible that the here used anti-CRY1 antibody, which is built on the full-length *Drosophila* CRY, recognizes all aphid CRY proteins. To test this, we performed Western Blots with *Drosophila* and aphid CRYs. Aphid CRY1 (64 kDa) and CRY2 (both CRY2-1 and CRY2-2 are 59 kDa) differ in weight by about 5 kDa that should make it possible to differentiate them in Western Blots. In *D. melanogaster* wild-type flies, we found the expected CRY1 band at 62 kDa, which was absent in *cry* knock-out mutants (Figure 1). In *A. pisum*, we found two bands, one at 64 kDa and the second at 65 kDa. No band was visible at 59 kDA, which is the expected size of CRY2-1 and CRY2-2. Therefore, we assume either that the antibody does not recognize aphid CRY2-1 and CRY2-2 but only aphid CRY1 or that CRY2 is not expressed to a detectable amount at the protein level. The band at 64 kDa is exactly at the expected size of aphid CRY1 and the band at 65 kDa might be a stable and fully phosphorylated form of CRY1 (see discussion). Since we can still not completely rule out that the *Drosophila* CRY1 antibody does recognizes several aphid CRYs, in the following, we will just talk about aphid CRY.

After exposing the insects to 5 days of constant light, the band of *Drosophila* CRY1 did almost disappear, which is expected since *Drosophila* CRY1 is highly unstable in presence of light (Emery et al., 2000). However, the two bands of aphid CRYs remained unchanged, suggesting that aphid CRYs are not degraded by light.

### The Two PER Antibodies Stain the Same Cells on the Aphid Brain

**Figure 2:**
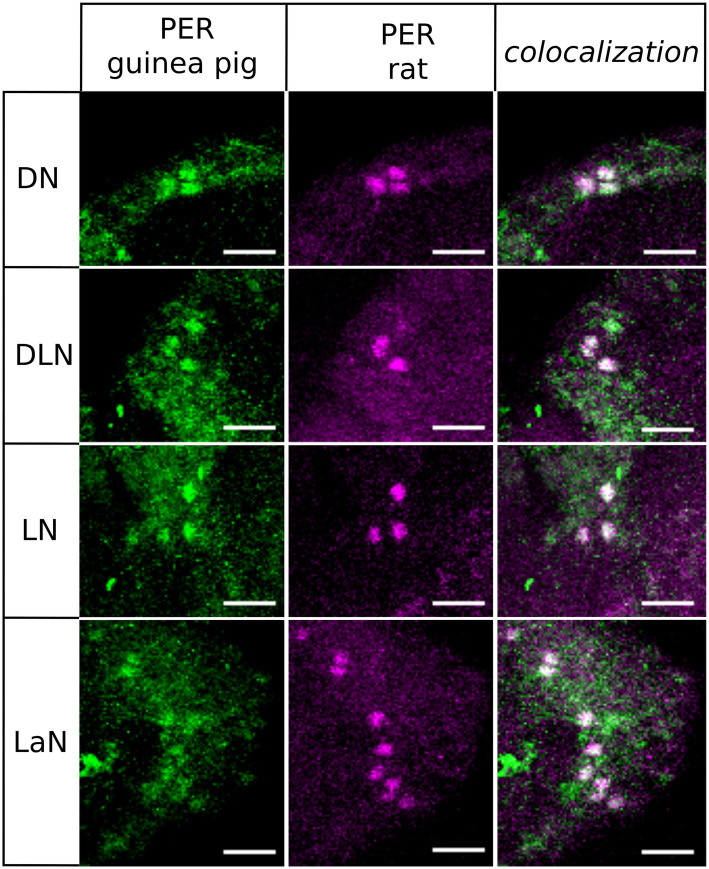
Co-immunostainings with anti-PER raised in guinea pig and anti-PER raised in rat. We show that all clusters of clock neurons co-localize the two antibodies (overlay of five to eight confocal stacks, scale bar: 15 μm).

We performed whole-mount immunostainings of seven complete aphid brains with two different PER-antibodies: one against 50AA in the C-terminal and the other against 100AA in the C-terminal of aphid PER. The results (Figure 2) revealed that the guinea pig antibody recognizes more cells compared to the rat antibody and has a higher background staining. We decided to regard only the cells that were labeled by both antibodies as “real” clock neurons and to perform the subsequent stainings with the apparently more specific rat antibody. In summary, we conclude that the antibodies reliably recognize aphid PER. PER-positive neurons were present in four clusters of neurons in each hemisphere, which were located in the Dorsal (DN), Dorsolateral (DLN), Lateral (LN) protocerebrum, and in the Lamina (LaN). We called them DN, DLN, LN, and LaN clock neurons, respectively (Figure 2). In the following, we will describe the four clusters of clock neurons in detail.

### PER and CRY Co-localize in the Dorsal, Lateral Central Brain, and in the Lamina of the Optic Lobe

**Figure 3:**
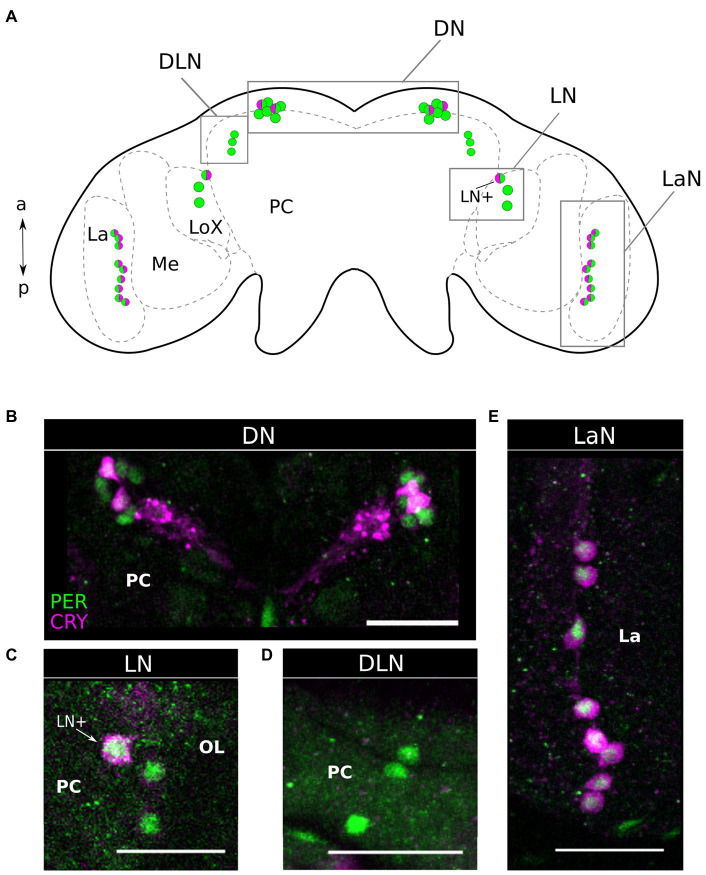
Period and Cryptochrome are expressed in four clusters of neurons. **(A)** Scheme of the aphid brain with the neurons of interest. The PER expressing neuron is in green while the CRY expressing neurons are in magenta. LA, Lamina; ME, Medulla; LoX, Lobula complex; and PC, Protocerebrum. **(B–E)** *via* co-immunohistochemistry using anti-PER and anti-CRY1, we show that these clock proteins are expressed in four clusters of neurons. **(D)** The DLN is the only cluster of PER positive cells, in which no CRY signal is detectable. Dorsal Neurons (DN), Dorso Lateral Neurons (DLN), Lateral Neurons (LN), Lamina Neurons (LaN). OL, Optic lobe; PC, Protocerebrum; and La, Lamina. Scale bar: 30 μm.

**Figure 4:**
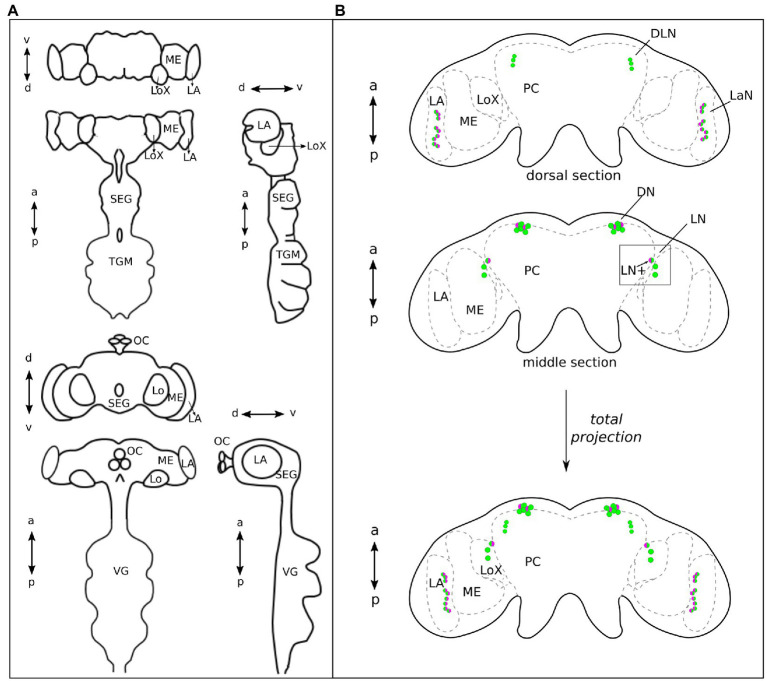
The central brain of Aphids lays on the same plane as the ventral ganglia, while in Drosophila it almost forms a 90° angle with the ventral ganglia. **(A)** Aphid and Drosophila brains, in the upper and lower part of the panel, respectively, are shown in frontal, dorsal, and lateral views, respectively. This explains why the dorsal view of the aphid brain corresponds to the frontal view of the Drosophila brain, in the organizations of the main neuropiles. **(B)** Green, PER-positive neurons and magenta, CRY-positive neurons. The aphid brain in the dorsal view is divided into two sections: anterior and middle. The ventral section is not shown because no clock neurons are present. At the bottom, the total projection of the two sections is shown. The DN cluster is located most anteriorly, while the LaN cluster is most posterior. On the other hand, the DLN cluster is most dorsal and the LN cluster together with the LaN cluster most ventral. a, anterior; p, posterior; d, dorsal; v, ventral; LN+, lateral neuron expressing PER and CRY; ME, Medulla; LoX, Lobula complex; LA, lamina; SEG, Subesophageal ganglion; TGM, Thoracic ganglionic mass; and VG, Ventral Ganglia of *Drosophila* that correspond to the TGM of aphids.

Double labeling of PER and CRY in 37 aphid brains collected at ZT21 revealed that CRY co-localized with PER in subsets of neurons in most of the four clock neuron clusters (Figures 3, 4B). In the following, we describe them from anterior to posterior. Please note that the *Drosophila* and aphid nervous system are different in respect of brain orientation (Figure 4A). While the brain of *Drosophila* is almost perpendicular to the ventral nervous system, the brain of aphids forms more or less a line with the ventral nervous system. This means that neurons that are located in the anterior brain of *Drosophila* are located ventrally in the aphid brain. Similarly, cells in the dorsal *Drosophila* brain are in the anterior aphid brain. In spite of these differences, the lateral and dorsal neurons of both insects appear to be largely comparable.

The aphid dorsal DN cluster was the most anteriorly located clock neuron cluster. It consisted of 6–8 PER-positive neurons, of which usually two strongly co-express CRY (Figures 3A,B, 4B). In 24% of the brains (9 out of 37), we found two further cells co-expressing CRY weakly. Thus, about half of the DN may co-express CRY. In this respect, the aphid DN cluster largely resembles the DN_1_ cluster of *Drosophila*, of which also half of the neurons co-express CRY (Yoshii et al., 2008; Menegazzi et al., 2020).

Slightly more lateral to the DNs, we found a cluster of three PER-positive neurons of which none co-expressed CRY (Figures 3A,D, 4B). Fuchikawa et al. (2017) showed that the honeybee, *A. mellifera,* possesses a cluster of PER-positive neurons located in the same brain region, suggesting that the two clusters may be identical. Therefore, we call these PER-positive cells DLN as in *A. mellifera*.

On the lateral edge of the protocerebrum in front of the lobula complex and adjacent to the medulla, we localized the LN clock neuron cluster. It consisted of 3–4 PER-positive cells, of which always one neuron strongly co-expressed CRY (Figures 3A,C, 4B). In 49% (19 out of 37) also other LN cells showed weak CRY staining in the cytoplasm. From their location, the aphid LN resembles the LN_1_ of honeybees (Fuchikawa et al., 2017; Beer et al., 2018) and the LN_d_ of fruit flies (Schubert et al., 2018), but they consist of less neurons. The single-aphid LN that strongly co-expressed CRY was often slightly detached from the others suggesting that it may be a separate cell. To clearly distinguish it from the other LN clock neurons, we will call it LN+ in the following (Figures 3A,C, 4B).

The LaN cells were located in the most posterior and dorsal region of the lamina. They consisted of 6–10 neurons that all co-expressed PER and CRY (Figures 3A,E, 4B).

**Figure 5:**
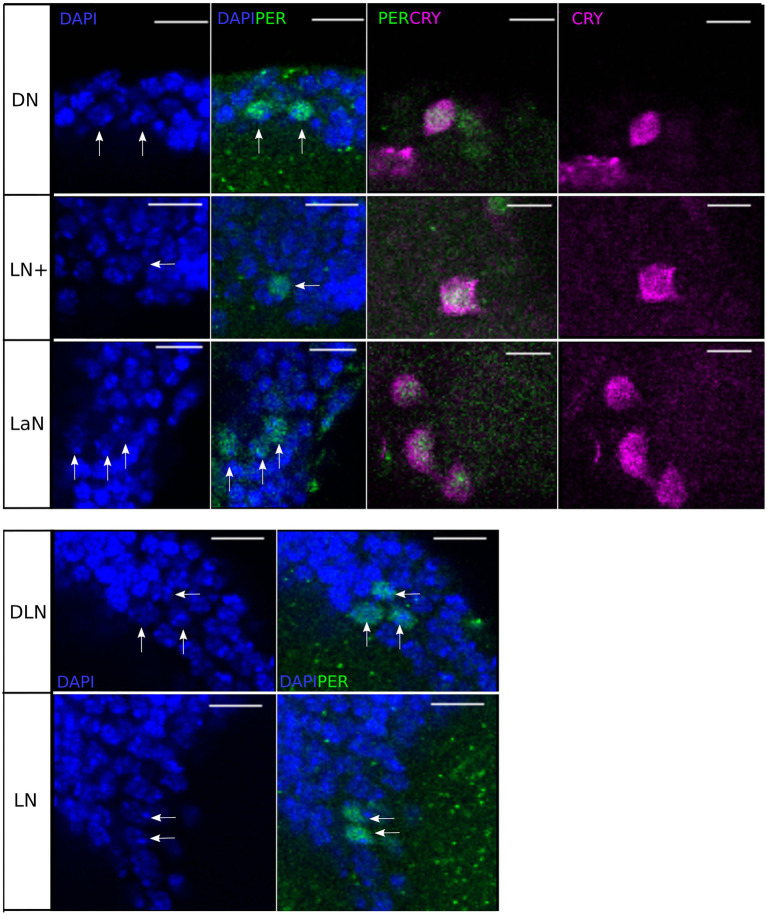
Single stacks of co-immunostainings with anti-PER, anti-CRY, and DAPI at ZT21. DAPI and anti-PER co-localize in the nuclei of all clock neurons. Moreover, in the clusters co-localizing anti-CRY and anti-PER (DN, LN+, and LN), anti-PER is nuclear while anti-CRY is mainly cytoplasmic. Scale-bar: 10 μm.

**Figure 6:**
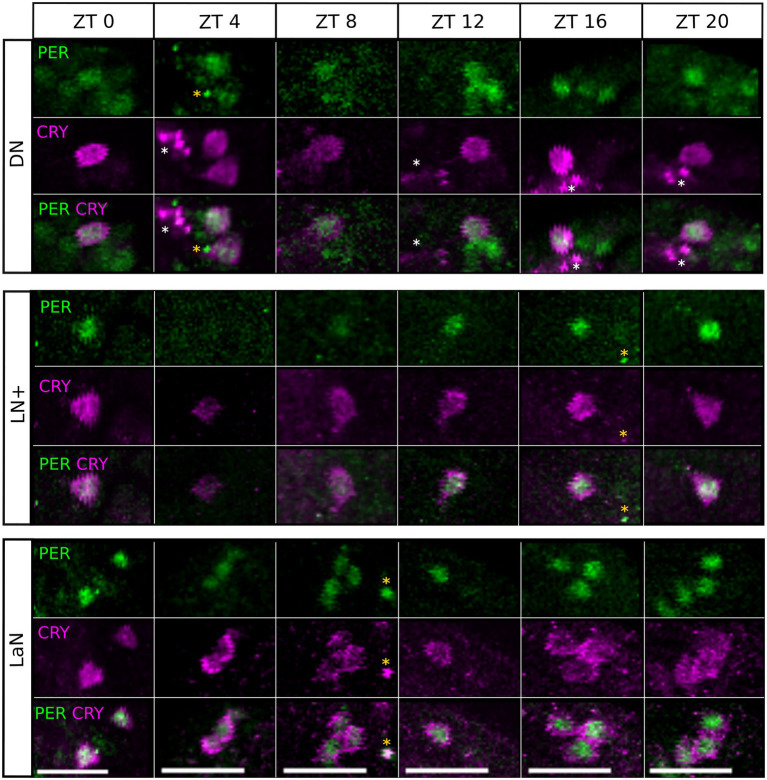
Single stacks of co-immunostainings with anti-CRY and anti-PER at different time points. The location of PER and CRY remains similar in all time points and depends on the cluster. PER signal is mainly nuclear, with some labeling in the region surrounding the nucleus. In the LaN, CRY signal is mainly cytoplasmic while in the DN and LN+, it partially localizes also in the nucleus. White asterisks: CRY arborizations. Orange asterisk: unspecific signal. Scale bar: 20 μm.

To check the precise localization of PER and CRY within the individual cells, we co-stained the PER-immunolabeled brains with DAPI (at ZT21). DAPI intercalates in double-stranded DNA and therefore stains specifically the nuclei of all cells in the aphid brain. We found that PER shares the nuclear space with DAPI and can therefore be regarded as completely nuclear (Figure 5). The localization of CRY, instead, is predominantly cytoplasmic, with some nuclear labeling (Figure 5 DN, LN+, LaN). The nuclear localization of PER and cytoplasmic localization of CRY was not only true at ZT21, but also at all other times (Figure 6). PER was always localized in the nucleus and sometimes detectable around it, but never covered the entire cytoplasmic area (e.g., Figure 6, ZT4 DN or ZT0 LN+). This result strongly reminds the situation in honeybees and mice, where PER did also not accumulate to detectable levels in the cytoplasm prior to its translocation into the nucleus (Smyllie et al., 2016; Fuchikawa et al., 2017).

### PER and CRY Expression Oscillate in a Daily Manner in All Clock Cells

**Figure 7:**
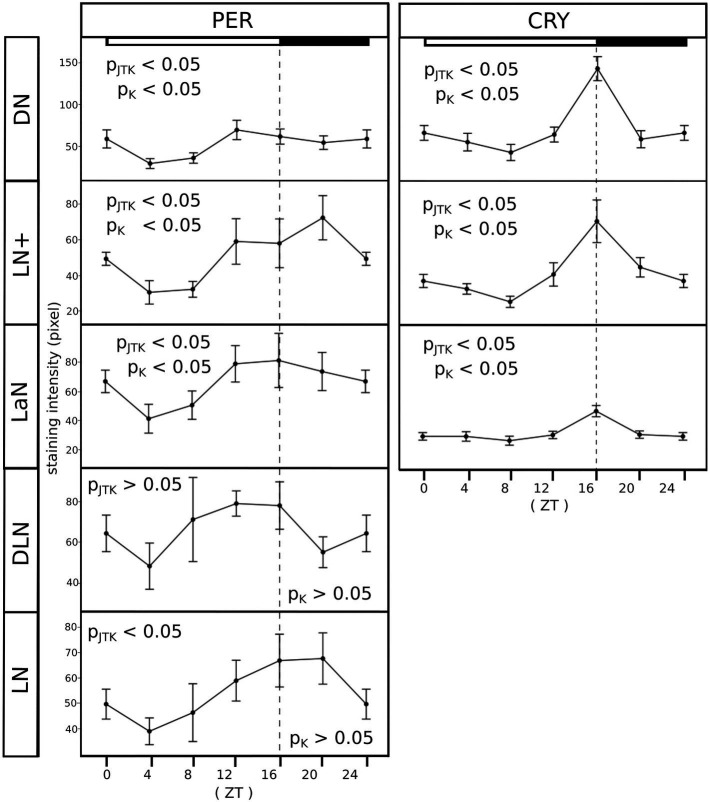
All clusters oscillate in a daily manner. PER and CRY staining was evaluated at six time points (ZT, Zeitgeber time). Each point of the curves represents the average between the intensities calculated in 7–10 brains. For clarity, ZT0 was plotted again at ZT24. The X-axes depict the time points (ZT) at which aphids were collected and the Y-axes depict the staining intensity. Error bar: standard error. P_JTK_, *p*-value resulting from the JTK_CYCLE analysis and *p*_k_, *p*-value resulting from the Kruskal–Wallis test. The exact *p-*values are reported in Table 1. The white/black bars show the illumination regime at which aphids where entrained (white bar: 16 h of light. Black bar: 8 h of darkness).

An important characteristic of clock cells is that clock protein expression oscillates, leading to different amounts of protein depending on the time of the day. To verify that the same happens in the clock neuron clusters identified in the aphid, we measured PER- and CRY-immunostaining intensity throughout the day. By visual inspection and verification *via* JTK_CYCLE analysis, PER and CRY expression oscillated in a daily manner in all clusters (Figure 7, *p*_JTK_ = *p-*value resulting from the JTK_CYCLE analysis, Table 1 for the exact values) except for the DLN cluster. In addition, the difference between time points in the curves was also tested with a Kruskal–Wallis test, which revealed statistically significant differences in PER staining only in those clusters expressing both proteins, namely the DN, LN+, and LaN (Figure 7, p_K_ = *p* value resulting from the Kruskal–Wallis analysis, Table 1 for the exact values). These results suggest that the presence of CRY might influence the turnover of PER and increase the amplitude of PER cycling. The CRY staining intensity was perfectly synchronized between clusters, being maximal at the end of the day and dropping down at the beginning of the night. PER staining oscillated instead with different phases depending on the cluster. In the DNs, the maximum staining (ZT12) was advanced compared to the LN+ (ZT20), resembling the phase differences among DNs and LNs found in *Drosophila* when the insect was exposed to summer conditions with a light-dark cycle of about 16:8 (Menegazzi et al., 2013).

**Table 1:** All clusters oscillate in a daily manner, except for the DLN.

	PER					CRY		
	DN	LN+	LaN	DLN	LN	DN	LN+	LaN
*p*-value Kruskal-Wallis	0.022	0.034	0.042	0.12615	0.097621	0.002	0.003	0.023
Adj. p JTK_CYCLE	0.01455	0.017568	0.016143	0.138182	0.013049	0.012023	0.000933	0.022645

The difference between the time points in every cluster was tested with the Kruskal-Wallis test, while the rhythmicity of the resulting curves was tested with the JTK_CYCLE algorithm. In red, we marked the only values >0.05 (not significant).

### CRY Neurons Show Widespread Arborizations in the Central Brain of Aphids

**Figure 8:**
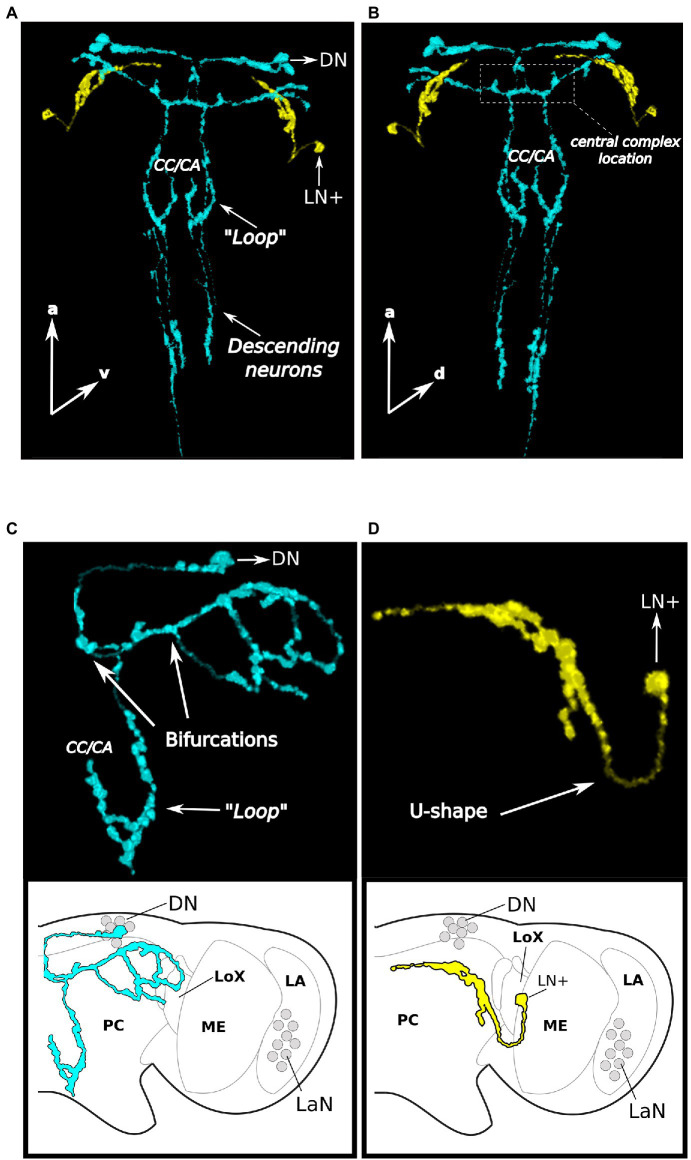
CRY neurons arborize widely in the aphid protocerebrum and descend to the thoracic ganglia. Two brains are reported as examples in **(A)** and **(B)**, first example, the arborizations are shown in a dorsal and ventral view, respectively, and the posteriorly descending neurons are visible in **(C)** and **(D)**, second example, only the DN arborizations (without descending neurons) and the LN+ arborizations are shown, respectively. **(A–C)**. The 3D reconstruction of the CRY positive DNs (light-blue) shows that they project toward the pars intercerebralis, turn toward the central complex and then bifurcate twice. Some fibers run laterally while others run posteriorly, form a “Loop” in the region corresponding to the corpora cardiaca/allata complex and then descend further. **(A,B,D).** The fiber starting from the LN+ neuron (in yellow) makes a U-shape-turn and projects anteriorly. CC/CA, Corpora cardiaca/corpora allata; PC, Protocerebrum; ME, Medulla; LA, Lamina; LoX, Lobula complex; and LaN, Lamina Neurons.

If the clock neurons identified so far are part of a network, one would expect them to be connected among each other by their own axons or by interneurons. Performing the co-immunostainings with anti-PER and anti-CRY, we noticed that CRY was often present in the axons and therefore, we decided to trace the CRY-positive fibers to understand where they project in the aphid brain. Since PER was only present in the nucleus, the 3D reconstruction could not be performed on the neurons which express PER only. While we could not see CRY positive fibers originating from the LaN, the other CRY-positive cells arborized widely in the brain and innervated the central, lateral, dorsal, and ventral protocerebrum (Figures 8A,B).

The CRY-positive LN+ neuron of each hemisphere was rather easy to trace. It formed a U-shaped turn and ran toward the most anterior and median dorsal region of the brain (yellow stained fiber in Figures 8A,B,D), where it terminated posteriorly and slightly ventrally of the DN cluster. It did not cross the midline of the brain and did not touch the fibers originating from the two CRY-positive DN, although it came close to them.

The two CRY-positive DNs had a more complex arborization pattern. Since the fibers arising from the two neurons largely overlapped, we were not able to separate them. Therefore, we describe the projection pattern of the two neurons together (shown in blue in Figures 8A–C). They first projected toward the pars intercerebralis, which is located in the dorsal midline of the brain. There, the fibers coming from both hemispheres came very close to each other and might even cross to the other side. They turned posteriorly toward the central complex (Figures 8A,B), which they surrounded. They passed the central complex, turned laterally and bifurcated two times (Figure 8C). In the first bifurcation, one branch ran posteriorly and descended toward the ventral nervous system (see below) and the other branch ran laterally toward the lateral protocerebrum. On the halfway before reaching the edge of the protocerebrum, the laterally running branch bifurcated for the second time and the two newly arising branches surrounded the lateral lobe from the dorsal and ventral side, respectively (Figure 8C).

At the height of the corpora cardiaca/allata complex (Steel, 1977), the descending projections of the CRY-positive DNs form a dorsal “*Loop*” into the direction of the corpora cardiaca/allata (Figures 8A–C) before descending further toward the ventral nervous system (Figures 8A,B).

### Anti-PDH Did Not Reveal Any Staining in the Aphid Brain

To reveal the arborizations of further clock neurons, we applied anti-PDH, an antiserum that unraveled the projections of clock neurons in a wide variety of insects (Homberg et al., 1991; Stengl and Homberg, 1994; Helfrich-Förster, 1995, 2005; Helfrich-Förster et al., 1998; Sehadová et al., 2003; Závodská et al., 2003; Shiga and Numata, 2009; Vafopoulou and Steel, 2012; Ikeno et al., 2014; Shafer and Yao, 2014; Stengl and Arendt, 2016; Beer and Helfrich-Förster, 2020). In 10 brains immunostained with anti-PDH, we could not detect any labeling.

## Discussion

As a first step toward the understanding of circadian clock involvement in photoperiodism, we characterized here the neuronal network of the circadian clock in the strongly photoperiodic pea aphid. In the following, we will compare our results on the aphid clock with results gained for other insects, and at the end, we will discuss the possibility that the aphid clock makes connections to the photoperiodic system.

### The Pea Aphid Circadian Clock Localizes in the Protocerebrum and in the Optic Lobes

We found that PER and CRY are expressed in four clusters of neurons located in the dorsal protocerebrum (DN and DLN), in the lateral protocerebrum proximal to the optic lobes (LN) and in the distal region of the optic lobes (LaN). These are the exact locations where the transcript of *per* was localized by Barberà et al. (2017). A similar location of PER and CRY expressing neurons has been shown also in other insects (Helfrich-Förster, 2005; Numata et al., 2015; Beer and Helfrich-Förster, 2020), although there are obvious differences regarding the anatomical location of the LNs and the presence of the LaNs.

The differences in the LN location among diverse insects will be reviewed in the following. In larger insects with pronounced optic tract (optic stalk), the LNs are located in the proximal optic lobe, close to the medulla, and anterior to the lobula complex. Smaller insects, such as fruit flies and aphids, have no evident optic tract. Their optic lobes form more or less a continuum with the central brain and, consequently, the LNs are located between the optic lobes and the central brain, which looks as if they are located in the lateral protocerebrum. Nevertheless, in almost all insects investigated so far, the LNs send neurites into the so-called accessory medulla, a small neuropil at the anterior base of the medulla, and in most insects, the PDF-positive LNs play a prominent role among these neurons (reviewed in Helfrich-Förster et al., 1998; Stengl and Arendt, 2016; Beer and Helfrich-Förster, 2020). The accessory medulla is regarded as circadian pacemaker center or master clock of many insects. Per definition, a circadian pacemaker center or master clock can control rhythmic activity even in absence of other clock neurons. The PDF-positive LNs appear to fulfill the role of a master clock in the flies, *D. melanogaster* and *Protophormia terraenovae* and in the cockroach *Rhyparobia maderae*, although, in the latter insect, these cells are not called LNs but accessory medulla neurons (Petri et al., 1995; Reischig and Stengl, 2003; Shiga and Numata, 2009; Stengl and Arendt, 2016; Helfrich-Förster, 2017). Furthermore, the LNs are very prominent and they may also work as master clocks in the honey bee, *A. mellifera* (Fuchikawa et al., 2017; Beer et al., 2018), the ant, *Camponotus floridanus* (Kay et al., 2018), and the beetle *Pachymorpha sexguttata* (Frisch et al., 1996).

The main differences with respect to these insects are that in aphids, the LNs are much reduced in number. In addition, the ventral group of the LNs appears to be absent. In *D. melanogaster*, the ventral group of LNs consists of eight neurons that express PDF (Helfrich-Förster, 1995). In the aphid brain, we did not see any PDF staining and found only three LNs, which underlines the notion that the ventral LNs could be completely absent. Moreover, in *Drosophila,* the dorsal group of lateral neurons, the LN_d_s, consists of six neurons, of which three are strongly CRY positive and three are usually CRY negative (Yoshii et al., 2008). Of the three aphid LNs, one neuron strongly expresses CRY, but in 49% of the cases the others are weakly CRY positive. This is very similar in *Drosophila*, where the three CRY-negative LN_d_s sometimes weakly express CRY. Among the 6–8 aphid DNs, two strongly express CRY and two further cells express CRY weakly. Very similarly, about half of the ~15 *Drosophila* DN_1_s express CRY (Yoshii et al., 2008).

In the cricket *Gryllus bimaculatus*, the master clock appears instead to localize not in the LNs, but rather in the lamina neurons of the distal optic lobes (Tomioka and Chiba, 1986; Tomioka, 2014). In particular, the latter neurons express PER and CRY and are located both in the distal lamina and in the chiasma between medulla and lamina. This is what we observe in aphids, too. The lamina neurons are also located in between of the two neuropiles and may thus be homologous to one of the two clusters of cricket lamina neurons. CRY immunostaining is also present in a lamina-associated structure, the lamina organ, in the cockroaches *R. maderae* and *Blaberus craniifer* (Fleissner et al., 2001). Furthermore, PER-positive neurons have been described in the lamina of the cockroaches *Blatella germanica*, *B. bisignata* (Wen and Lee, 2008), and the beetle *P. sexguttata* (Frisch et al., 1996), and these appear homologous to the PDF-positive lamina clock neurons of *R. maderae* (Giese et al., 2018). However, at least in the Madeira cockroach, these cells are not necessary for maintaining behavioral rhythmicity, but instead couple the light cycle of the external environment with the master clock in the accessory medulla (Fleissner et al., 2001). In other insects investigated so far (flies, bees, ants, moths, and butterflies), clock neurons were not located in the lamina (Helfrich-Förster, 2005; Kay et al., 2018; Beer and Helfrich-Förster, 2020).

The great majority of the so far investigated insects possesses clock neurons in the dorsal protocerebrum (Frisch et al., 1996; Helfrich-Förster et al., 1998; Helfrich-Förster, 2005; Beer and Helfrich-Förster, 2020), and at least in the moth *Antheraea pernyi* and the Monarch butterfly *Danaus plexippus*, the PER-positive DNs are more important in controlling circadian activity rhythms than the LNs (Sauman and Reppert, 1996a,b; Sauman et al., 2005; Reppert, 2006). At present, nothing is known about the location of the master clock in aphids, but anatomically the aphid clock neurons are similar to those of other insects. They resemble those of bees and ants concerning the DNs, those of crickets, cockroaches, and beetles concerning the LaNs, and those of fruit flies concerning the co-localization of PER and CRY in subsets of LNs and DNs.

In summary, we can say that the PER/CRY-positive neurons of aphids appear to be homologous to the circadian clock neurons described in other insects, but also show differences regarding cluster size and organization. Therefore, we cannot draw conclusions on the function of the different clock cells by comparing them with other insects. Future studies are necessary to reveal the functional role of the clock neurons in the aphid clock system.

### The *Drosophila* CRY1 Antibody Most Likely Detects CRY1 in the Pea Aphid

The *A. pisum* genome encompasses three *cryptochrome* genes: the *Drosophila*-type *cry*1 and two copies of the mammalian-type *cry*2 (Cortés et al., 2010). Our *Drosophila* CRY1 antibody appears to detect mainly CRY1 in the aphid brain. This conclusion is based on the following findings (1) In Western Blots, no band with the predicted size of CRY2-1 and CRY2-2 was detected, (2) CRY2s as transcriptional repressors should be mainly nuclear, but we found only very little CRY in the nucleus, (3) CRY2 should cycle in parallel to PER, but aphid CRY cycled in parallel to the light-dark cycle, which is expected from CRY1, and (4) the CRY antibody stained all arborizations of the LN and DN as is expected of the photoreceptor CRY1, but not of CRY2. All this indicates that we have stained CRY1 and not CRY2. Nevertheless, we cannot exclude that CRY2 is present in the same cells working in the core clock mechanism as in mosquitos, butterflies, and crickets (Zhu et al., 2005; Kutaragi et al., 2018). We also do not know the identity of the second CRY band at 65 kDa. CRY1 is a complex molecule that possesses several putative phosphorylation sites (Liu and Zhang, 2016; Damulewicz and Mazzotta, 2020). Although we do not see a second CRY band in the Western Blot of *Drosophila* CRY1, we cannot exclude that phosphorylated aphid CRY1 is very stable and can be seen in the Western Blot. Alternatively, the band at 65 kDa might stem from a still unknown isoform of CRY1. Future studies have to reveal the identity of the second CRY band.

### PER and CRY Oscillate in a Daily Manner

Clock proteins should show daily oscillations in abundance so that they can deliver rhythmicity to the cell, e.g., make neurons fire in a daily manner. We found that aphid PER and CRY met this criterion. PER and CRY expression oscillated in a daily manner in all neuron clusters. However, the difference in PER staining between time points in the curves, tested with Kruskal-Wallis, was statistically significant only in those clusters that expressed both proteins (DN, LN+, and LaN). This feature suggests that the presence of CRY influences the turnover of PER and may amplify the difference in the protein amount during the day. PER staining intensity oscillated with different phases depending on the cluster: the DNs and LaNs peaked ~4–6 h earlier than the LNs. This phase relationship largely resembles the phase relation between DNs and LNs in *Drosophila* when the insect was exposed to long-day conditions with 16 h or even longer days (Menegazzi et al., 2013).

Regarding CRY cycling, our experiments yielded additional interesting results. First, the CRY oscillations occurred in high synchrony in all neurons, peaking at ZT16 and having the trough at ZT8. This strongly suggests that they are synchronized by light-dark cycles. Second, CRY was not degraded by light, but its amount increased until the light switched off, which is the opposite of what happens in *Drosophila’s* clock neurons. Western Blots confirmed the stability of CRY under constant light. The light stability of aphid CRY may be explained by the lack of the *jetlag* gene in the aphid genome (Cortés et al., 2010). Jetlag is a key player in the light-dependent degradation of CRY1 and TIM in flies (Peschel et al., 2009). Obviously, in aphids, the CRY1 oscillations are synchronized to the light-dark cycles by a different molecular mechanism than in flies, in which CRY1 is not degraded but its gene transcription or translation is rather stimulated by light. In the circadian clock of crickets, CRY1 is not degraded by light either (Tokuoka et al., 2017). Similar to aphids, crickets possess *cry*1 and *cry*2 in their genome, and the two *cryptochromes* appear involved in a second negative feedback loop that is independent of the *tim*/*per* feedback loop (Tokuoka et al., 2017). Light perception in the crickets relies solely on the compound eye with a green-light sensitive opsin receiving light signals (Komada et al., 2015), and the two cricket *cryptochromes* appear be involved in the regulation of light entrainment of the circadian clock (Kutaragi et al., 2018). A similar clock mechanism may be possible for the aphid, because the clock gene sets of crickets and aphids are rather similar and both species belong to the hemiptera and are thus evolutionary related (Beer and Helfrich-Förster, 2020). Future studies will have to show what role CRYs have in the aphid and the cricket clock to elucidate whether these species share a rather similar clock mechanism or just share some features.

### PDF Is Most Likely Absent in Aphids

In many insect clocks investigated so far, PDF plays a pivotal role in modulating circadian rhythms and synchronizing the clock neurons with each other. Given its presence in the cell bodies and in the axons of clock neurons, PDF immunohistochemistry is widely used to characterize un-explored insect clock networks. However, there are a few insects lacking PDF-positive cells in the lateral brain/optic lobe [the sphinx moth *Manduca sexta* (Wise et al., 2002)] or seem to lack the *pdf* gene completely [the red flour beetle *T. castaneum* (Li et al., 2008)]. Not all species of the order hemiptera lack PDF, for example, PDF is present in the cicada *Meimuna opalifera* (Sato et al., 2002) and also in different species of Heteroptera, such as the *Gerris paludum*, *Rhodnius prolixus*, and *Riptortus pedestris* (Závodská et al., 2003; Vafopoulou and Steel, 2012; Ikeno et al., 2014). However, the *pdf* gene could not be found in aphids so far (Huybrechts et al., 2010; Li et al., 2020). This suggests that this neuropeptide has been lost in certain specific clades, e.g., the clade *Sternorrhyncha* (plant lice), which is a sister clade of the *Heteroptera* (true bugs) and *Auchenorrhyncha* (cicada), or even only in specific subordinate groups of these clades (e.g., the Aphidoidea within the *Sternorrhyncha*). We conducted our immunohistochemical experiments using an anti-PDH antibody that has been shown to recognize PDH and PDF peptides throughout the panarthropoda, including onychophorans, tardigrades, crustaceans, and insects (Mayer et al., 2015), and we could not stain PDF-positive cells in the aphid brain, suggesting that the *pdf* gene has truly been lost in aphids.

Nevertheless, there is also another explanation: Sequences coding for aphid PDF may have diverged during evolution so much that neither a homology-based BLAST search, nor an antibody recognizing PDFs is able to identify aphid PDF. This happened in the beetle *T. castaneum*, for which no PDF gene sequence could be identified (Li et al., 2008), but *T. castaneum* PDF has undergone significant changes especially in the C-terminal amino acid sequence, therefore only an elevated comparative analysis of several beetle genome sequences and RNAseq assemblies could identify the predicted PDF sequence, while the G protein coupled receptor of the PDF type was immediately identified (Veenstra, 2019). Similarly, the PDF receptor has been annotated for *A. pisum* (Li et al., 2013) and recently also for the cowpea aphid, *Aphis craccivora* (Li et al., 2020), while in both insects the PDF peptide was not spotted. Possibly, the amino acid sequences of aphid PDF diverge so much that they are not recognized by the antibody. Still another possibility is that *pdf* got lost, but the receptor gene persisted since the receptor responds to other neuropeptides that have overtaken the role of PDF in the aphid clock network. Future studies on other clock-associated peptides in the aphid and the aphid PDF receptor may help to distinguish these possibilities.

### The CRY Staining Reveals Putative Connections of the Clock Neurons to the Aphid Photoperiodic System

As already stated by Barberà et al. (2017, 2019), the clock gene expressing DNs of *A. pisum* are in an optimal position to communicate with the neurosecretory cells (NSCs) in the pars intercerebralis and lateralis. Steel and Lees (1977) found that these brain regions are essential for controlling photoperiodic responses in *Megoura viciae*. Especially NSCs in the pars intercerebralis appeared to produce parthenogenesis promoting factors called viginoparins under long-day conditions. Recent studies showed that ILPs are most likely the proposed viginoparins that are produced in the NSC cells of *A. pisum* (Barberà et al., 2019; Cuti et al., under revision). ILPs are released from the corpora cardiaca/allata complex into the circulation and additionally from neurosecretory fibers terminating in the neighborhood of the ovaries keeping the aphids in the parthenogenetic state (see footnote 1). Here, we show that CRY-positive fibers from the clock neurons not only run into the pars intercerebralis but additionally toward the corpora cardiaca/allata complex making the proposed communication between the circadian clock and the photoperiodic system very likely. It is tempting to speculate that the CRY-positive clock neurons convey photoperiodic signals from the circadian clock to the NSCs and the corpora cardiaca/allata complex that then release key hormones affecting reproduction. In line with this hypothesis, CRYs have been shown to mediate photoperiodic responses in the cricket *Modicogryllus siamensis* (Ueda et al., 2018). In nature, cricket development is prolonged under short-day conditions. Ueda et al. downregulated the two cricket *cry* genes and found reduced photoperiodic responses to long-day conditions while the photoperiodic responses to short-day conditions were enhanced. This led to a strongly delayed development or a complete lack of adult emergence (Ueda et al., 2018). The authors concluded that CRYs are involved in measuring day length in crickets. Future work has to reveal whether aphid CRYs play a similar role and whether the aphid clock neurons indeed communicate with the NSCs controlling seasonal reproduction.

## Data Availability Statement

The raw data supporting the conclusions of this article will be made available by the authors, without undue reservation.

## Author Contributions

FC performed the experiments with contribution of KB to the immunohistochemical stainings, of PD to the Western Blots, and of PC to the preparation and staining of the aphid brains throughout the 24-h day. TY generated the PER antibody in guinea pigs, CH-F and DM supervised the study, and FC, KB, and CH-F wrote the manuscript. All authors provided comments and approved the manuscript.

### Conflict of Interest

The authors declare that the research was conducted in the absence of any commercial or financial relationships that could be construed as a potential conflict of interest.
